# Supplementary material for: The Current Developments in Medicinal Plant Genomics Enabled the Diversification of Secondary Metabolites’ Biosynthesis
Source: Int J Mol Sci. 2022 Dec 14;23(24):15932. doi: 10.3390/ijms232415932 (PMC9781956; doi:10.3390/ijms232415932)
Supplement: Supplementary file 1 [file ijms-23-15932-s001.zip › ijms-2069319-Supplementary Materials.pdf]

Supplementary Table S1. Plant genomics completed by Sanger sequencing technology.

Species	Genome size (Mb)	Coverage	References
G. max	1100	8.0X	[173]
B. distachyon	272	9.4X	[174]
R. communis	320	4.6X	[175]
Zea mays	2300	6.0X	[176]
Sorgum bicolor	730	8.5X	[177]
Physcomitrella patens	511	9.0X	[178]
Carica papaya	372	3.0X	[179]
Vitis vinifera	487	8.4X	[180]
Populus trichocarpa	485	7.5X	[181]
Oryza sativa indica	466	4.0X	[182]
O. sativa japonica	420	6.0X	[183]
A. thaliana	125	-	[184]

Supplementary Table S2. Plant genomics completed by second-generation sequencing technology.

Species	Genome size (Mb)	Genome Coverage	Scaffolds	Contigs	N50	Sequence method	Assembly method	References
Asclepias syriaca	236.77 Mb	80.4x	221855	268318	1505	Illumina	Platanus v. 1.2.1; SCUBAT v. 2015-12-17	[185]
Panax ginseng	2980	307x	9845	240888	22539	Illumina	SOAPdenovo2	[186]
Chrysanthemum seticuspe	2721.84	96x	354212	961201	7793	Illumina	SOAPdenovo v. 2.0.4; SSPACE v. 2.0; GapCloser v. 1.10; GapFiller v1-10	[187]
Megacarpaea delavayi	883.81	150x		763815	65480	Illumina	Platanus v1.2.4	[188]
Sinapis alba	449.668	96x	12410	30825	98426	Illumina	discovarDeNovo v. 51464	[189]
Mesua ferrea	614.35	180x	503130	587971	55831	Illumina	MaSuRCA version 3.3.1	[190]
Platycodon grandiflorus	680.178	480.2x	4816	75346	58279	Illumina HiSeq	SOAPdenovo2 v. 2.04; Celera Assembler v. 8.1; GARM v. 0.7.5; CAP3 v. 02-10-2015	[51]
Benincasa hispida	912.95	50.0x	1791	14936	144985	Illumina	AllPaths v. ALLPATHS-LG-44837	[191]
Dioscorea rotundata	456.675	100x	21	34429	22084	Illumina HiSeq 2500	Allpaths-LG v. r49856, SSPACE Premium v. 2.3	[192]
Vernicia fordii	1,176.32	100x	20614			Illumina Hiseq	SOAPdenovo2 r240, SSPACE v1.2	[58]
Lablab purpureus		194x	118976	135039	32223	Illumina HiSeq	SOAPdenovo v. 2-2.04	[193]
Mimosa pudica	557.202	413x	97892	119356	27447	Illumina HiSeq	Platanus v. 1	[194]
Gnetum montanum	4070	302x	142000	475170	25019	Illumina HiSeq	SOAPdenovo	[195]
Hydrangea macrophylla	2178	108x	3779	15791	1400000	Illumina	Platanus, FALCON v.1.8.8	[196]
Pterocarya stenoptera	955.601	300x	124315	211800	37442	Illumina	MaSuRCA v. 2.3.3; SOAPdenovo2 v. 2.04	[197]
Lavandula angustifolia	870	100x	84291			Illumina HiSeq	FERMI, OPERA, GapCloser	[198]
Ocimum basilicum	2067.62	65x	17105		48300	Illumina NovaSeq 6000	Supernova v2.1.1	[199]
Origanum majorana	760.89	65x	8763		35950	Illumina NovaSeq 6000	Supernova v2.1.1	[199]
Origanum vulgare	630.04	65x	13832		26280	Illumina NovaSeq 6000	Supernova v2.1.1	[199]
Pogostemon cablin	1916.69	355x	41698		202492	Illumina HiSeq	SOAPdenovo v. 2.04	[200]
Rosmarinus officinalis	1013.85	65x	23035		21820	Illumina NovaSeq 6000	Supernova v2.1.1	[199]
Moringa oleifera		398x	22329	29972	45268	Illumina HiSeq	SOAPdenovo v. 2-2.04	[193]

Nelumbo nucifera	817.268	100x	2341	53811	44440	Illumina HiSeq, 454	AllPaths v. 40102; ALLMAPS v. June-2017	[201]
Osmanthus fragrans	733.5	100x		145	1595720	Illumina HiSeq	Falcon v0.3.0, Arrow	[202]
Gastrodia elata	1060.98	150x	3768	35404	97233	Illumina	AllPaths v. 44080	[203]
Phytolacca americana	933	83x	847766		35208	Illumina	Discovar De Novo	[204]
Abies alba	18167	152x	37192295	45280944	2477	Illumina	SOAPdenovo, BESSTv2.2.5	[205]
Cydonia oblonga	488.422	108x		303932	2435	Illumina	SOAPdenovo v. 2	[206]
Chiococca alba	558	301x	3518	15168	60292	Illumina	ALLPATHS-LG	[54]
Citrus reticulata	344.273	200x	67725	77693	97052	Illumina HiSeq	Platanus v. 1.2.4	[207]
Santalum album	220.961	90x	12821	29203	25151	Illumina HiSeq	SPAdes v. 3.6.1	[208]
Aquilaria sinensis	783.887	55.67x	14276	47303	60210	Illumina HiSeq	HiRise v. May-2019	[209]
Boehmeria nivea	344.617	256x	12775	32476	24523	Illumina HiSeq	Platanus v. 2.23	[210]
Polygonum cuspidatum	2565	147x	948118	1078298	2769	Illumina HiSeq	SOAPdenovo	[211]
Dracaena cambodiana	1064.43	89x	2379659	2640704	1859	Illumina HiSeq 2500	SOAPdenovo	[212]
Alnus glutinosa	611.874	326x	167345	211045	9564	Illumina HiSeq	Platanus v. 1	[194]

Supplementary Table S3. Medicinal plant genome completed by third-generation sequencing.

Species	Genome size (MB)	Genome Coverage	Scaffolds	Contigs	N50	Sequence method	Assembly method	References
Andrographis paniculata	269.408 Mb	100.0x	257	1278	388864	PacBio RSII	CANU v. 1.5	[59]
Strobilanthes Cusia	865.49 Mb	130x		1602	4329179	Illumina Hiseq, Oxford Nanopore, Hi-C	Caun, Falcon, WTDGB	[52]
Atriplex hortensis	964	68x		3183	1114696	Illumina, Nanopore	Canu v.1.7.1	[213]
Allium sativum	16559.4	188x	18350	121677	193988	PacBio Sequel	FALCON v. 1.0	[49]
Mangifera indica	392.98	240x	252	421	3579047	Illumina, PacBio	Canu v.1.8	[214]
Pistacia vera	671.28	374x	1865	2327	714252	Illumina, PacBio	PBJelly v. 15.8.24	[215]
Annona muricata	656.77	676x	949	2066	784561	Illumina, PacBio	SOAPdenovo2	[216]
Apium graveolens	3,332.57	232.22X	4,863	9,496	790578	PacBio Sequel I, Illumina HiSeq, 10X Genomics	FALCON, Quiver	[217]
Coriandrum sativum	2118.68	271x	6186	9936	604128	Pacbio, Illumina		[218]
Panax notoginseng	2359.97	110x	179913	616542	16420	PacBio, Illumina	SOAPdenovo(v2.04)	
Asparagus setaceus	710.152	156.0x	665	1,879	1367059	Oxford Nanopore GridION	Canu v. 1.8	[219]
Aloe vera	129300	48x	7545697		14560	Illumina, Nanopore	wtdbg2 v2.0.0	[220]
Artemisia annua	1792.86	180x	39400	190477	20144	454, PacBio, Illumina	Newbler v. Oct-2014	[47]
Conyza canadensis	426.383	248.0x	357	859	1620735	PacBio, Illumina, Hi-C	CANU v. 1.8; Proximo v. 2.0	[221]
Erigeron breviscapus	1,430	384x	1812	18973	140946	Illumina, PacBio RSII, Hi-C	wtdbg	[222]
Mikania micrantha	1790.64	134.0x	2815	4414	1353263	Illumina, PacBio	Canu v.1.6, FALCON v. 1.2.0, FALCON-Unzip v. 1.1.1	[223]
Betula platyphylla	430.49	122x		1550	751259	Illumina, PacBio	pb-asse ly (v0.0.2), wtdbg2 (v2.1)	[224]
Corylus mandshurica	367	309x		81	14849403	Illumina, Nanopore	NextDenovo (v.2.1)	[225]
Lithospermum erythrorhizon	367.41	94x		2465	314306	Oxford Nanopore, Illumina	Abyss v2.1.5, DBG2OLC hybrid assembler	[225]

Erysimum cheiranthoides	177.181	190x	223	931	1657946	Illumina, PacBio	FALCON v. 2017-06-07	[226]
Isatis indigotica	293.865	142.71x	809	1198	1176212	PacBio Sequel	Canu v. 1.6; wtdbg v. 1; Quickmerge v. 0.2	[227]
Chimonanthus praecox	695.36	346.35x	763	1623	2190841	Illumina HiSeq, 10X Genomics, PacBio	FALCON	[228]
Chimonanthus salicifolius	853.434	532.0x	689	1788	2121821	PacBio, Illumina, 10X Genomics, Hi-C	FALCON v. 0.3; Pilon v. 1.16; FragScaff v. 140324.1; LACHESIS v. 1	[63]
Cannabis sativa	876.148	100x	221	1052	1959202	PacBio, Nanopore, Illumina	minimap, miniasm2, pilon, BWA	
Lonicera japonica	843.2	100x	9	919	2148893	Oxford Nanopore	CANU 1.8, SMARTdenovo, pilon	[229]
Casuarina equisetifolia	301.458	546.93x	2936	6546	466697	Illumina HiSeq; PacBio	SOAP-denovo v. 2.04.4; FALCON v. 1.8.2; DISCOVAR v. 52488	[230]
Tripterygium wilfordii	348.533	793.34x	321	467	4362171	llumina HiSeq, PacBio	FALCON v. 3.1	[3]
Kochia scoparia	711.357	300.0x	19671	61599	20773	Illumina, PacBio	MeCat v. April-2018	[231]
Cuscuta australis	262.63	97.6x	103	218	3625894	PacBio, Illumina HiSeq	Canu v. Oct-2016	[232]
Cuscuta campestris	476.792	60x	6907	27832	48808	PacBio, Illumina	DISCOVAR de novo r52488, SSPACE-Standard v3.0, SSPACE-Longread v1.1, GapFiller v1.10	[233]
Cucurbita argyrosperma	228.82	151x	920	1481	463388	Illumina, PacBio	Platanus, DBG2OLC	[234]
Luffa cylindrica	656.19	371x	332	480	8800239	Illumina HiSeq, PacBio	FALCON, Quiver, BWA-MEM, LACHESIS	[235]
Momordica charantia	302.992	84x	193	211	9902508	PacBio Sequel, HiSeq2500	Canu v. 1.7; ALLMAPS	[236]
Sechium edule	608.17	151x	103	473	8400000	Illumina, Nanopore	Canu	[237]
Siraitia grosvenorii	469.5	180x		4128	432384	PacBio, Illumina	FALCON, Pilon	[238]
Trichosanthes anguina	919.8	172.1x	69	202	20110000	Nanopore, Illumina HiSeq, Hi-C	Canu, SMARTdenovo	[239]
Dioscorea dumetorum	485.115	207x		924	3190870	Illumina, Nanopore	Canu v1.8	[240]

Trichopus Zeylanicus	713.407	83.0x	22601	25896	288821	Illumina HiSeq, PacBio Sequel	MaSuRCA v. 3.2.3	
Diospyros lotus	907	65X		3073	1060344	Illumina, PacBio	Falcon	[226]
Rhododendron simsii	528.637	270x	552	899	2274689	Illumina, PacBio	SMARTDENOVO v. 1.0	[241]
Eucommia ulmoides	1100	93x	29348	132188	15969	Illumina, PacBio	Platanus assembler v1.2.1; SSPACE v2.0; PBJelly2 v14.1.14; BWA v0.7.16a	[60]
Abrus precatorius	347.23	140.0x	160	344	11837218	Illumina, PacBio	FALCON v. 2018	[242]
Arachis hypogaea	2551.68	260x	29	32721	213557	Illumina, PacBio, Bionano	DeNovoMAGIC2 v. 2	[243]
Dalbergia odorifera	638.26	637.83x	384		5920000	PacBio, Illumina HiSeq, Hi-C	Falcon v2.0.5, Quiver v5.0	[244]
Pisum sativum	3920	294x	24623	218010	37931	Illumina, PacBio	SoapdeNovo2, SSPACE 2.0	[245]
Senna tora	526.357	146.0x	444	738	3966958	PacBio RSII; PacBio Sequel	FALCON-Unzip v. June-2018	[56]
Spatholobus suberectus	798.47	624x	816	1967	2052247	PacBio Sequel, Illumina	FALCON v. 3.1	[246]
Castanea mollissima	785.53	121x		2707	944461	Illumina, PacBio	SMARTdenovo	[247]
Gelsemium elegans	335.13	160x	545	738	10238528	Oxford nanopore, Illumina	Canu v1.7.1, WTDBG v1.2.8	[248]
Hypericum perforatum	373.65	653x	448	755	1409424	Illumina, PacBio	FALCON assembler2	[57]
Callicarpa americana	506.362	81x	328	965	7510543	PacBio, Illumina HiSeq, Hi-C	Canu v1.7	[249]
Salvia bowleyana	462.44	232x	238	1154	1180000	Illumina, PacBio	MECAT, Canu	[62]
Salvia splendens	809.16	63x	1525	2051	2340816	PacBio, Illumina	CANU v. 1.5	[250]
Scutellaria baicalensis	386.674	173.37x	114	950	880642	Illumina HiSeq X Ten, PacBio Sequel	HI-C v. Mar-2018; 10XGenomics v. Mar-2018	[53]
Scutellaria barbata	353.0	130x			2500000	PacBio, Oxford Nanopore	CANU (v1.7), SMARTdenovo, Pilon (v1.22)	[17]
Cinnamomum kanehirae	730.416	85.0x	2150	6084	498920	PacBio	FALCON v. 0.5.0	[251]
Litsea cubeba	1,325.68	150.0x	1,515	3,682	613688	PacBio Sequel	FALCON v. 3.1	[252]

Liriodendron chinense	1742.42	87x	3710	4183	2204006	Illumina, PacBio, Bionano	FALCON v. 0.3.0	[253]
Magnolia biondii	2232	190x	9510	15615	269114	Illumina, PacBio	Canu v. 0.136, Miniasm v. 0.337, Wtdbg v. 1.1.006, Flye v. 2.3.338, SMARTdenovo 1.0.0	[50]
Bombax ceiba	895	350x	2759	3145	1023644	Pacbio, Bionano	FALCON v0.3.0, PBJelly 2, Pilon	[254]
Paris polyphylla	701800	98.59x	15306712	78957901	1814	BGISEQ-500, 10X Genomics Chromium	SOAPdenovo v. LR	
Toona sinensis	596.35	333x		789	1525641	Illumina HiSeq, Nanopore, Hi-C	CANU v. 1.8, SMARTdenovo v1.0, Racon v1.3.3, Medaka v0.7.1, Pilon v1.23, bowtie2-2.3.5, HiC-Pro version 2.8.0_devel, Juicer v1.5, 3D de novo assembly (3D-DNA) v180922	[255]
Broussonetia papyrifera	386.83	311.73x	345	3851	171170	Illumina, Bionano	ALLPATHS-LG, SSPACE, BioNano Genomics,	[256]
Ficus carica	333.44	74.0x	511	905	823517	PacBio Sequel	FALCON-Unzip v. v0.5	[257]
Ficus hispida	369	250x		4194	492000	Illumina, PacBio	CANU, SMARTdenovo	[258]
Ficus microcarpa	403 M	250x		5536	908000	Illumina, PacBio	FALCON, SMARTdenovo	[258]
Morus alba	336.47	147.0x	249	519	3089666	Oxford Nanopore, Illumina HiSeq, Hi-C	canu v. December-2018; smartdenovo v. December-2018; juicer v. December-2018; 3d-dna v. December-2018	[259]
Psidium guajava	443.756	115.0x	44	73	15807569	PacBio Sequel	FALCON v. 0.3	[170]
Euryale ferox	725.2	230x		451	4750000	Illumina, Nanopore	Canu assembler	[260]
Forsythia suspensa	737.47	113.1x		1213	7333645	Nanopore, ONT, Promethion	WTDBG2 v. 2018-09-24	[261]
Dendrobium huoshanense	1285 Mb	352x			598000	Illumina HiSeq, PacBio	Smart denovo	[262]

Averrhoa carambola	320.43	546x			4219540	Nanopore, Illumina HiSeq, Hi-C	Canu v1.7	[263]
Paeonia suffruticosa	13793	67x		499810	499337	PacBio	Falcon v1.8.7	[264]
Papaver somniferum	2715.53	239.0x	34381	65344	1773300	llumina HiSeq, PacBio, 10X Genomics	DeNovoMAGIC v. 3.0; Falcon v. 3.1	[30]
Passiflora edulis	1,332.18	222.43x			3,100,000	Oxford Nanopore, Illumina NovaSeq, Hi-C	NextDenovo,Smartdenovo	[265]
Piper nigrum	761.22	275x	45			BioNano, PacBio, Illumina	Canu, FALCON, BWA-MEM	[48]
Antirrhinum majus	520	174x	620	1645	733198	Illumina, PacBio	CANU, SSPACE	[266]
Coix lacryma-jobi	1731.46	238.6x	1233	8757	3121225	Illumina HiSeq, PacBio RSII, PacBio Sequel	HERA v. 2018	[267]
Setaria viridis	397.03	130.0x	9	45	19521898	Illumina, Nanopore	Hybrid assembly (miniasm, minimap2, racon) v. NOVEMBER-2019	[268]
Aegiceras corniculatum	906.63	237x			7100000	Illumina, PacBio	CANU v. version 2.0	[269]
Eriobotrya japonica	760.10	131x		597	5020000	Illumina, Nanopore	Canu v1.4, SMARTdenovo	[270]
Potentilla micrantha	327	192x	2674	33026	16235	Illumina, PacBio	ALLPATHs-LG, PBJelly	[271]
Prunus salicina	284.209	255x	272	272	1777944	Illumina, PacBio	FALCON v. 0.3.0	[272]
Rosa chinensis	513.854	80.0x	55	88	22201688	Illumina HiSeq, Hi-C	TIL-R v. Rev: 523; CANU v. 1.6	[273]
Gardenia jasminoides	535.679	60x	81	940	1028321	Oxford Nanopore, Hi-C	Canu v. 1.8	[61]
Ophiorrhiza pumila	440.319	308x	13	36	18486819	PacBio RS II, PacBio Sequel, Illumina HiSeq	Canu v. 1.6	[7]
Poncirus trifoliata	264.9	220x	152	809	842800	Illumina, PacBio	Falcon v2018.03.12-04.00	[274]
Populus alba var. pyramidalis	464.516	350x	17797	55988	26535	Illumina HiSeq, PacBio	Platanus, SSPACE-LongRead v1-1, PBJelly v15.8.24	[275]

Acer truncatum	633.28	531.6x	34	1453	773169	Illumina, PacBio, Hi-C	Falcon, FALCON-Unzip, Quiver, Pilon, FragScaff	[55]
Xanthoceras sorbifolium	505.79	375x	267	2002	645453	Illumina, PacBio	Falcon v. 0.7.0	[276]
Argania spinosa	670.097	236x	75327	78992	43178	Illumina, PacBio	MaSuRCA v. 2018	[277]
Selaginella tamariscina	300.729	50x	1391	2143	228918	PacBio	FALCON v. 0.3	[278]
Datura stramonium	1288.64	30.85x	30384	31084	56707	Illumina, PacBio	SparseAssembler v. Aug-2015, DBG2OLC v. Aug-2016, Canu v. 2.0, OPERA-LG v. 2.0.6	[279]
Solanum tuberosum	716.17	70x	12		13367893	Illumina, Nanopore	Canu v. 1.8	[280]
Vitis amurensis	603.559	320x	3040	5873	344503	Illumina, PacBio	Platanus v. 1.2.4; DBG2OLC v. 2016-11-23	[281]
Coptis chinensis Franch	1.15 Gb	100x	108		806,550	Illumina, Nanopore, Hi–C	Canu (v1.6), SMARTdenovo (v1.5)	[2]

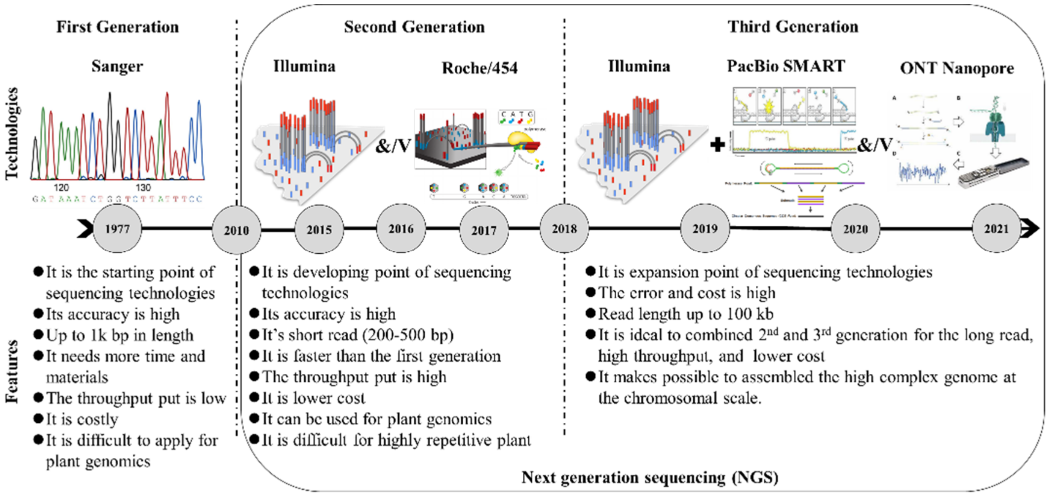

Supplementary Figure S1. The revolution of genome sequencing technologies.
